# Metabolic Characterization of *Hyoscyamus niger* Ornithine Decarboxylase

**DOI:** 10.3389/fpls.2019.00229

**Published:** 2019-02-27

**Authors:** Tengfei Zhao, Changjian Wang, Feng Bai, Siqi Li, Chunxian Yang, Fangyuan Zhang, Ge Bai, Min Chen, Xiaozhong Lan, Zhihua Liao

**Affiliations:** ^1^Key Laboratory of Eco-Environments in Three Gorges Reservoir Region (Ministry of Education), Chongqing Engineering Research Centre for Sweet Potato, TAAHC-SWU Medicinal Plant Joint R&D Centre, School of Life Sciences, Southwest University, Chongqing, China; ^2^Tobacco Breeding and Biotechnology Research Center, Yunnan Academy of Tobacco Agricultural Sciences, Key Laboratory of Tobacco Biotechnological Breeding, National Tobacco Genetic Engineering Research Center, Kunming, China; ^3^College of Pharmaceutical Sciences, Key Laboratory of Luminescent and Real-Time Analytical Chemistry (Ministry of Education), Southwest University, Chongqing, China; ^4^TAAHC-SWU Medicinal Plant Joint R&D Centre, Xizang Agricultural and Husbandry College, Nyingchi of Tibet, China

**Keywords:** biosynthesis, *Hyoscyamus niger*, ornithine decarboxylase, polyamine, tropane alkaloids

## Abstract

Ornithine decarboxylase (ODC) catalyzes ornithine decarboxylation to yield putrescine, a key precursor of polyamines, and tropane alkaloids (TAs). Here, to investigate in depth the role of ODC in polyamine/TA biosynthesis and to provide a candidate gene for engineering polyamine/TA production, the ODC gene (*HnODC*) was characterized from *Hyoscyamus niger*, a TA-producing plant. Our phylogenetic analysis revealed that HnODC was clustered with ODC enzymes of plants. Experimental work showed *HnODC* highly expressed in *H. niger* roots and induced by methyl jasmonate (MeJA). In the MeJA treatment, the production of both putrescine and *N*-methylputrescine were markedly promoted in roots, while contents of putrescine, spermidine, and spermine were all significantly increased in leaves. By contrast, MeJA did not significantly change the production of either hyoscyamine or scopolamine in *H. niger* plants. Building on these results, the 50-kDa His-tagged HnODC proteins were purified for enzymatic assays. When ornithine was fed to HnODC, the putrescine product was detected by HPLC, indicating HnODC catalyzed ornithine to form putrescine. Finally, we also investigated the enzymatic kinetics of HnODC. Its *K*_m_, *V*_max_, and *K*_cat_ values for ornithine were respectively 2.62 ± 0.11 mM, 1.87 ± 0.023 nmol min^-1^ μg^-1^ and 1.57 ± 0.015 s^-1^, at pH 8.0 and at 30°C. The HnODC enzyme displays a much higher catalytic efficiency than most reported plant ODCs, suggesting it may be an ideal candidate gene for engineering polyamine/TA biosynthesis.

## Introduction

**FIGURE 1:**
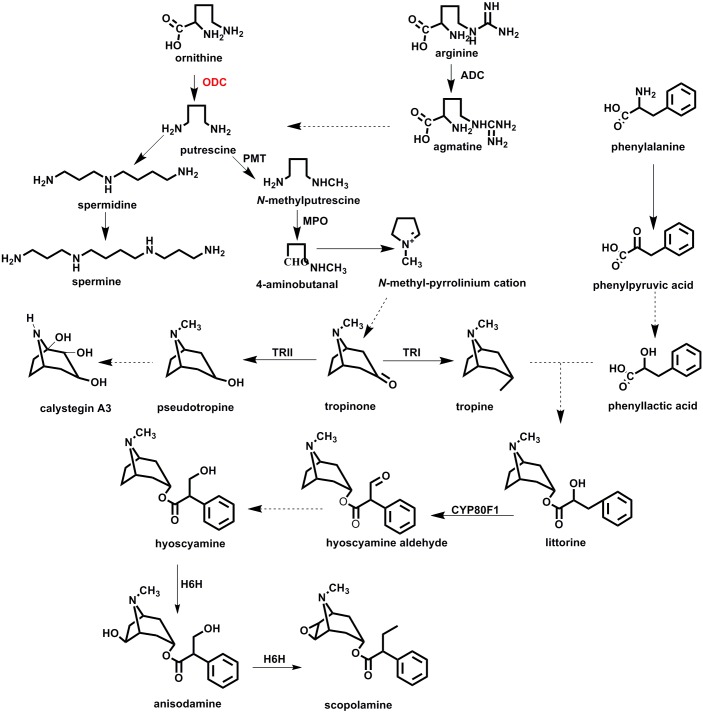
The biosynthetic pathway of tropane alkaloids in plant species of Solanaceae. ADC, arginine decarboxylase; ODC, ornithine decarboxylase; MPO, *N*-methylputrescine oxidase; TRI, tropinone reductase I; TRII, tropinone reductase II; CYP80F1, littorine mutase; H6H, hyoscyamine 6β-hydroxylase.

Polyamines, including putrescine, spermidine, and spermine, are involved in many important biological processes of plants, such as their growth, development, and adaption to biotic and abiotic stresses (Kusano et al., 2008; Kusano and Suzuki, 2015; Aloisi et al., 2016). Moreover, putrescine is essential for the synthesis of polyamines and putrescine-derived alkaloids (Jirschitzka et al., 2012), because forming putrescine is the first step in the polyamines biosynthetic pathway (Figure 1), providing a ley precursor for spermine and spermidine (Kusano and Suzuki, 2015). Putrescine can become methylated to form *N*-methylputrescine, a key intermediate compound of nicotine and pharmaceutical tropane alkaloids (TAs) (Biastoff et al., 2009). Among medicinal plants belonging to the Solanaceae family, such as *Hyoscyamus niger*, *Atropa belladonna*, and *Datura species*, all produce pharmaceutical TAs, including hyoscyamine and scopolamine which are widely used as anticholinergic reagents.

In many plants, putrescine is synthesized directly from ornithine by ornithine decarboxylase (ODC), or indirectly from arginine by arginine decarboxylase (ADC) (Figure 1). That putrescine is ubiquitous in plants is perhaps not surprising, given its crucial functioning in plant metabolism (Tiburcio et al., 1997). Tissue localization of ornithine/arginine decarboxylases suggests that ODC could be the main enzyme responsible for the synthesis of putrescine in plant roots (Wang et al., 2000; Delis et al., 2005). For solanaceous plants reported on to date, all their precursors of TAs are synthesized in roots and then transferred aboveground, to the plants’ aerial parts (Kanegae et al., 1994; Suzuki et al., 1999; Bedewitz et al., 2014). Hence, we may speculate that ODC rather than ADC participates in the biosynthesis of putrescine-derived alkaloids. To resolve this issue clearly requires further experimental investigation.

Ornithine decarboxylase is a rate-limiting enzyme in the biosynthesis of ornithine-derived metabolites (Bunsupa et al., 2016). More specifically, it tightly regulates putrescine production that dynamically affects the biosynthesis of polyamines and TAs (Figure 1). Because of its involvement in polyamine biosynthesis, the function of ODC has been well-studied and understood in animals and bacteria. With respect to plants, however, most studies of plant ODC, focused on its regulation on polyamine biosynthesis under stressful conditions (Akiyama and Jin, 2007; Pál et al., 2015; Krasuska et al., 2017). By tracing labeled ornithine, earlier work indicated that ornithine was used for TA biosynthesis (Hashimoto et al., 1989; Nyman, 1994), yet TA production was interrupted when *Hyoscyamus albus* plants were treated with difluoromethylornithine (DFMO), a specific inhibitor of ODC (Nyman, 1994). Although ODC undoubtedly participates in TA biosynthesis, its exact role in this process, especially at molecular and biochemical levels, remains largely unknown.

To our best knowledge, in plants, only the ODC proteins of *Nicotiana glutinosa* (NgODC) and *Erythroxylum coca* (EcODC) have been characterized for their enzymatic kinetics, by using purified proteins. The NgODC enzyme is associated with the biosynthesis of nicotine and has a very low catalytic activity (Lee and Cho, 2001); in contrast, the purified recombinant EcODC exhibits much higher catalytic efficiency than does NgODC (Table 1) (Docimo et al., 2012). Biochemical characterization of ODC proteins from tobacco and coca tree has fostered a richer understanding of their contribution to the regulation of nicotine and cocaine production, respectively. In solanaceous plants that produce TAs, *Datura stramonium* was the only species with its ODC gene (*DsODC*) cloned and characterized. In that work, *DsODC* was highly expressed *D*. *stramonium* roots and crude protein extracts from *E*. *coli* expressing *DsODC* demonstrated the ODC activity, but without a characterization of its kinetics (Michael et al., 1996). Therefore, it is valuable to further study the ODC roles in TA and polyamine biosynthesis in TA-producing plants.

**Table 1:** The kinetics parameters of three ornithine decarboxylases in plants.

Enzymes	Species	*K*_m_ (mM)	*V*_max_ (nmol min^-1^ μg^-1^)	*K*_cat_ (s^-1^)	*K*_cat_/*K*_m_ (M^-1^ s^-1^)	Reference
HnODC	*Hyoscyamus niger*	2.62 ± 0.11	1.87 ± 0.023	1.57 ± 0.015	599	This study
EcODC	*Erythroxylum coca*	0.395	0.248	0.18	465	Docimo et al., 2012
NgODC	*Nicotiana glutinosa*	0.56	0.0125	0.009.3	16.53	Lee and Cho, 2001


As a plant species well known for producing TAs, especially scopolamine, *Hyoscyamus niger* is also widely used for studying their biosynthesis. To date, several TA biosynthesis enzymes have been robustly characterized from *H*. *niger* as well as other plants species (Figure 1). These enzymes include putrescine *N*-methyltransferase (Liu et al., 2005; Kai et al., 2009b; Geng et al., 2018), tropinone reductase I (Nakajima et al., 1993b; Kai et al., 2009a; Qiang et al., 2016), tropinone reductase II (Hashimoto et al., 1992; Nakajima et al., 1993a,b), CYP80F1 (Li et al., 2006), and hyoscyamine 6β-hydroxylase (Matsuda et al., 1991; Li et al., 2012). Since their identification, the associated TA-biosynthesis genes have been applied to engineer TA biosynthesis in plants via the overexpression method (Zhang et al., 2004; Wang et al., 2011; Zhao et al., 2017). In this context, it is thus very important to distinguish those enzymes with higher catalytic activities to better facilitate the metabolic engineering of metabolite biosynthesis. However, very few plant ODC enzymes are ever studied in great detail for their enzymatic kinetics by using purified recombinant proteins. This gap in knowledge means that most results of ODC studied in TA-producing plants are preliminary.

To better understand ODC’s role in the biosynthesis of TAs and polyamines, the ODC gene (*HnODC*) was isolated from *H. niger*. Tissue profiling of *HnODC* was analyzed by using quantitative reverse transcriptase PCR. Furthermore, the expression patterns of *HnODC*, and TA-biosynthesis genes (*HnPMT*, *HnTRI*, and *HnH6H*) were investigated through MeJA treatment. Simultaneously, the ornithine-derive metabolites, including putrescine, spermidine, spermine, *N*-methylputrescine and two types of TAs (hyoscyamine and scopolamine), were analyzed. Finally, the purified recombinant HnODC was used to analyze its kinetics. Metabolic characterization of HnODC not only revealed its roles in the biosynthesis of TAs and polyamines, but also provided a candidate gene for potential use in TA engineering and polyamine production applications.

## Materials and Methods

### Plant Materials and MeJA Treatment

Mature seeds of *Hyoscyamus niger* were harvested from the medicinal plant garden of the Xizang Agricultural and Husbandry College of Nyingchi (Tibet, China) in August 2016, with their taxonomic identity confirmed by Professor Xiaozhong Lan. These seeds were germinated into plantlets in substrate composed of vermiculite:pindstrap moss:perlite (6:3:1) and grown at 25 ± 1°C under an 16 h-light/8 h-dark conditions. Once the plantlets reached 10 cm in height, their roots and leaves were respectively harvested for the tissue profile analysis of HnODC, *HnADC1*, *HnADC2*, and TA-biosynthesis genes, including *HnPMT*, *HnTRI*, and *HnH6H*. To determine whether MeJA influenced the expression levels of these genes and the metabolism of polyamines and TAs, the 10-cm-tall *H*. *niger* plants were treated with 100 μM of MeJA for 0, 1, 6, 12, and 24 h. Each duration had three replicate plants, from which the roots were harvested for RNA isolation and metabolite analysis. The leaves collected from same plants were used for metabolite detection. Plant material treated with a solution lacking MeJA for 24 h served as the control. Three or more independent plants per treatment were used in all analyses.

### Gene Cloning and Bioinformatics Analysis

Total RNA was extracted from the *H*. *niger* roots with RNAsimple Total RNA Kit, according to the manufacturer’s protocols (Tiangen Biotech, Beijing, China). 50–100 mg of material from each plant part was used to extract total RNA. The first-strand cDNA chain was synthesized by using a FastKing RT kit (Tiangen Biotech, Beijing, China). The prepared reaction mixture, with a total volume of 10 μl, contained 2 μl of buffer (DNase solution provided by the FastKing RT kit), 2 μg of total RNA, and ddH_2_O was incubated at 42°C for 3 min to remove any potential genomic DNA. Next, 2 μl of King RT buffer, 1 μl of FastKing RT Enzyme Mix, and 2 μl of FQ-RT Primer Mix were added into the reaction mixture; ddH_2_O was then also added to obtain the final volume of 20 μl. Then, this 20 μl of the RT reaction mixture was incubated at 42°C for 15 min and at 95°C for 3 min. All the cDNA samples were diluted 50 times with RNase-free water, after which 8 μl of cDNA solution served as templates for the RT-PCR.

A pair of gene-specific primers, HnODC-F and HnODC-R (Supplementary Table S1), was used to isolate the coding sequence of HnODC based on sequenced *H*. *niger* transcriptomes (data not published). Amplification reactions were performed in a final volume of 50-μl buffer containing 5 μl of TransTaq HiFi Buffer I (10×) with 20 mM MgSO_4_ (TransGen Biotech, Beijing, China), 4 μl of 2.5-mM dNTPs, 1 μl of each primer (10 mM), 2.5 U of TransTaq HiFi DNA polymerase, and 50 ng of template DNA. PCR conditions were set as follows: the templates were denatured at 94°C for 5 min, followed by 28 cycles (94°C for 30 s, 56°C for 30 s, and 72°C for 90 s), and finally incubated at 72°C for 8 min. PCR products were purified from 1.0% (w/v) agarose gel with a DNA purification kit (BioFlux, Hangzhou, China), then subcloned into pMD19-T for sequencing by using the following protocol: a reaction mixture that contained 1 μl of PMD19-T vector, 1 μl of DNA fragment, 3 μl of ddH_2_O, and 5 μl of Solution I was first prepared, then incubated at 16°C for 30 min and transformed into *E*. *coli*. The recombinant plasmid harboring HnODC was extracted from *E*. *coli* for sequencing. The sequence of HnODC was confirmed by sequencing it on a 3730 DNA Analyzer (Thermo Fisher Scientific, Waltham, MA, United States) using M13 forward and M13 reverse universal primers, via the Sanger sequencing approach. The BLAST analysis was performed online at the website^1^ (Johnson et al., 2008).Then multiple alignments were performed using the ClustalX bioinformatics program (Larkin et al., 2007). A phylogenetic tree was built by the neighbor-joining method in MEGA software v.5 (Tamura et al., 2011). Its bootstrapped values were generated from *n* = 1000 replicates to evaluate the accuracy of the phylogenetic construction.

### Gene Expression Analysis

To analyze the tissue profile of TA-biosynthesis genes *HnODC*, *HnADC1*, *HnADC2*, *HnPMT*, *HnTRI*, and *HnH6H*, their total RNAs were respectively extracted from the leaves and roots of *H*. *niger* plants (three biological replicates) according to the methods described above. Likewise, to analyze the expression patterns of these genes under the MeJA treatment, their RNAs were respectively extracted from the roots of plants treated with MeJA and control plants. After reverse-transcription into cDNAs, expression levels of the genes were analyzed by real-time quantitative PCR (qPCR), using the phosphoglycerate kinase gene (PGK) as an internal reference, by following the method of Li et al. (2014). The qPCR kits were purchased from BIO-RAD and the qPCR system was an IQ5 thermocycler (BIO-RAD, Hercules, CA, United States). The 2^-ΔΔCT^ method was used to calculate the relative gene expression levels (Livak and Schmittgen, 2001). At least three independent plants were used in this gene expression analysis. All primers were designed with the software tool Beacon Designer (Premier Biosoft International, Palo Alto, CA, United States), based on sequences publicly available from the NCBI GenBank database. Primer specificity was validated by melting profiles and consisted of a single product-specific melting temperature. The associated GenBank accession numbers are *HnODC* (MK169378), *HnPMT* (AB018572), *HnTRI* (D88156), and *HnH6H* (DQ812529). All the primers used are listed in Supplementary Table S1.

### Analysis of Alkaloids and Polyamines

*N*-methylputrescine and polyamines (putrescine, spermidine, and spermine) were extracted using the method described by Do et al. (2013). Root and leaf samples (1.00 g fresh weight, FW) from 24-h-MeJA-treated plants and corresponding control plants were homogenized respectively in liquid nitrogen, and then extracted in 4 ml of 0.2 N perchloric acid (PCA) at 4°C for 1 h. After centrifugation at 16,000 g at 4°C for 30 min, the supernatant was used to determine the plant content of polyamines. To do this, 1 ml of the supernatant was added with 10 μl of benzoyl chloride. After incubation at 37°C for 25 min in the dark, the benzoylzed polyamines were extracted with 2 ml of chloroform, dried with nitrogen flow, and then dissolved in 1 ml of methanol. From each ensuing sample, 20 μl were injected into HPLC for metabolite analysis (Flores and Galston, 1982). The detection methods used here were similar to those applied in the enzymatic assays described below. Tropane alkaloid content was quantified by adhering to previously described methodologies (Qiang et al., 2016; Zhao et al., 2017; Geng et al., 2018). For this, 200 mg of dry powder from each plant part was accurately weighed for alkaloid extraction and detection, with at least three independent plants used for metabolite detections.

### Protein Purification and Enzymatic Assay

The *HnODC* coding region was amplified by using two primers (HnODC-PF/HnODC-PR) containing a restriction site for *Bam*HI and *Sac*I (Supplementary Table S1). First, the PCR products of HnODC were purified and digested using *Bam*HI and *Sac*I. Then the purified HnODC coding region harboring the restriction sites of *Bam*HI and *Sac*I was inserted into a pET-28a+ vector, to generate the prokaryotic expression vector. The ligation mixture consisted of 1 μl of T4 DNA ligase, 1 μl of reaction buffer, 2 μl of linearized pET-28a+ vector, and 6 μl of purified HnODC. Next, the constructs were introduced to *E*. *coli* Rosetta for protein expression. Bacteria were cultured in an LB liquid medium with 50 mg/L of kanamycin and 34 mg/L of chloramphenicol at 37°C. When the OD value of bacterial cultures had reached 0.6, HnODC expression was induced by adding isopropyl β-D-1-thiogalactopyranoside (IPTG) at a final concentration of 0.5 mM. Bacteria were further cultured at 25°C for 7 h, then harvested for protein purifications. The recombinant His-tagged HnODC was purified via Ni^2+^-chelating resin columns using the same methods we reported in a previous study (Qiang et al., 2016).

To perform the enzymatic assays, we followed Docimo et al. (2012). To test the catalytic activity of HnODC, its purified form was tested in a 10-mM Hepes buffer (pH 8.0) containing 1 mM dithiothreitol and 1 mM pyridoxal phosphate (PLP) for 60 min, by using different concentrations of L-ornithine as the substrate. The products (putrescine) from enzymatic assays and authentic putrescine were benzoylated into benzoyl putrescine (as carried out by Geng et al., 2018). The benzoylated samples were used for the HPLC analysis or stored at -80°C for future use. All benzoylated samples were detected at 234 nm at an oven temperature of 30°C on a Shimadzu LC-20 HPLC system (Shimadzu Corp., Kyoto, Japan). A YMC-Pack ODS-A column was used (150 × 4.6 mmI. D.S-5 μm, 12 nm) and the flowing phage consisted of methanol:water (41:59) at a flow rate of 1 ml/min throughout the analysis. 20°μl of sample was injected for analysis. The Michaelis–Menten curve and Lineweaver-Burk plot of the HnODC enzyme were drawn to determine its *K*_m_ and *V*_max_ values, on which calculations of turnover rate (*K*_cat_) and catalytic efficiency (*K*_cat_/*K*_m_) were based.

## Results

### Molecular Cloning and Sequence Analysis of *HnODC*

**FIGURE 2:**
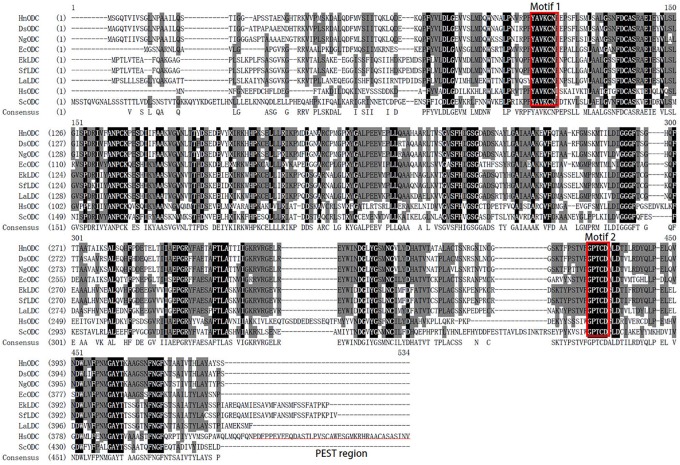
Multiple alignments of ornithine decarboxylases and lysine/ornithine decarboxylases. The motifs of YAVKCN and GPTCD are boxed in red. The PEST region in HsODC (human ornithine decarboxylase) is underlined. Gene Bank accession numbers are as follows: DsODC, *Datura stramonium* (CAA61121); EcODC, *Erythroxylum coca* (AEQ02350.1); HsODC, *Homo sapiens* (NP_001274119.1); NgODC, *Nicotiana glutinosa* (AAG45222.1); ScODC, *Saccharomyces cerevisiae* (DAA08982.1); LaLDC, *Lupinus angustifolius* (AB560664); SfLDC, *Sophora flavescens* (AB561138); EkLDC, *Echinosophora koreensis* (AB561139); HnODC, *Hyoscyamus niger* (MK169378).

**FIGURE 3:**
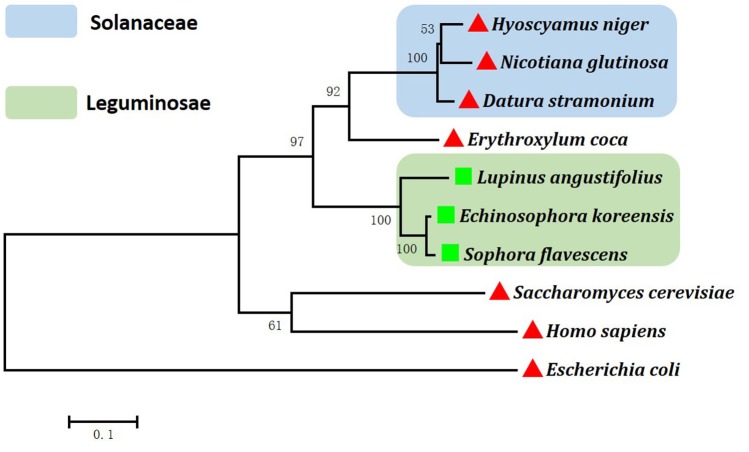
Phylogenetic analysis of ornithine decarboxylases and lysine/ornithine decarboxylases. 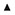 represents those ornithine decarboxylases confirmed with activity on the decarboxylation of ornithine. 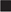 represent lysine/ornithine decarboxylases confirmed with activity on the decarboxylation of lysine/ornithine. The numbers on the phylogenetic tree are bootstrapped values (based on 1000 repeats). Gene Bank accession numbers are as follows: *Datura stramonium* (CAA61121); *Erythroxylum coca* (AEQ02350.1); *Homo sapiens* (NP_001274119.1); *Nicotiana glutinosa* (AAG45222.1); *Escherichia coli* (BAE77028.1); *Saccharomyces cerevisiae* (DAA08982.1); *Lupinus angustifolius* (AB560664); *Sophora flavescens* (AB561138); *Echinosophora koreensis* (AB561139); *Hyoscyamus niger* (MK169378).

**FIGURE 4:**
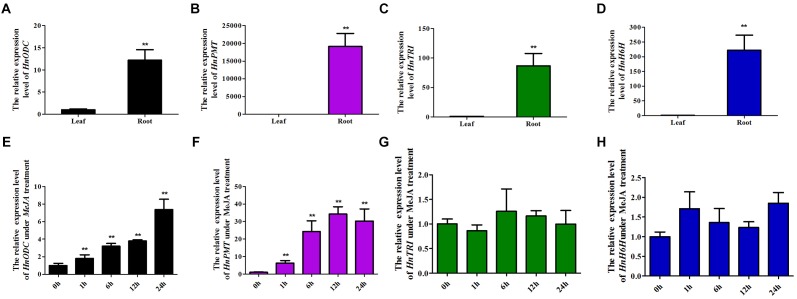
Tissue profiles of four TA-biosynthesis genes and their expression patterns in *Hyoscyamus niger* plants treated with MeJA for 0 to 24 h. Overall expression levels in roots and leaves of **(A)** *HnODC*, **(B)** *HnPMT*, **(C)** *HnTRI*, and **(D)** *HnH6H*. Expression of **(E)** *HnODC*, **(F)** *HnPMT*, **(G)** *HnTRI*, and **(H)** *HnH6H* according the duration of treatment with methyl jasmonate. Vertical bars are means ± standard errors (*n* ≥ 3). ^∗∗^indicates a significant difference at the level of *P* < 0.01 (*t* test).

The 1293-bp coding sequence of *HnODC* encoded a 430-amino-acid polypeptide (Supplementary Figure S1). The BLASTP analysis indicated HnODC belonged to the superfamily of type III PLP-dependent enzymes, and that it resembled the ODCs in GenBank. As the major binding site of α-DFMO (Coleman et al., 2003), the GPTCD motif was found at the C-regions in all of these ODC and LDC proteins (Figure 2). For HnODC, this motif was located at positions 372–376 (Figure 2), while the YAVKCN motif was at positions 90–95, and present in all the ODC and LDC sequences (Figure 2). Lysine in this motif was postulated to bind to the cofactor pyridoxal-5′-phosphate (Lee and Cho, 2001). But compared with the ODC enzymes of mammals, all plant ODC enzymes we found lacked the PEST regions required for constitutive and conditional degradation of ODC by the 26S proteasome system (Lee and Cho, 2001). Our phylogenetic analysis showed a distant evolutionary relationship between mammalian ODC enzymes and those from plants (Figure 3). All plant L/ODCs and ODCs occupied the same branch, which had two subgroups, and the L/ODCs are mainly from Leguminosae. Furthermore, HnODC showed closer evolutionary relationships with ODC proteins of Solanaceae plants and EcODC (Figure 4). *HnODC* has been deposited in GenBank (accession number MK169378).

### Gene Expression Analysis

Expression levels of *HnODC*, *HnADC1*, *HnADC2* and three TA-biosynthesis genes (*HnPMT*, *HnTRI*, and *HnH6H*) were detected in the roots and leaves of *H*. *niger* (Figure 4). However, all three genes—*HnPMT* (Figure 4B), *HnTRI* (Figure 4C), and *HnH6H* (Figure 4D)—were specifically expressed in the roots of *H*. *niger*, consistent with earlier reported findings (Hashimoto and Yamada, 1987; Hashimoto et al., 1992; Geng et al., 2018). Unlike the root-specific expression of those three TA biosynthesis genes, *HnODC* was expressed in both roots and leaves, but its expression level was still much higher in roots than leaves (Figure 4A). *HnADC1* was expressed in roots and leaves at similar level, and HnADC2 was expressed in roots and leaves with no significant difference (Supplementary Figure S2). However, these six genes responded differently to the MeJA treatment of *H*. *niger* plants (Figure 4): the transcript levels of *HnODC* (Figure 4E) and *HnPMT* (Figure 4F) were significantly increased, while the expression of *HnTRI* (Figure 4G), *HnH6H* (Figure 4H) and HnADC1/2 (Supplementary Figure S2) was unchanged. Across 1–24 h of the MeJA treatment, the expression of *HnODC* increased up to 6-fold that of the control, while that of *HnPMT* increased by 6–30 folds. These results indicated *HnODC* is mainly expressed in roots and induced by MeJA.

### Metabolite Analysis

**FIGURE 5:**
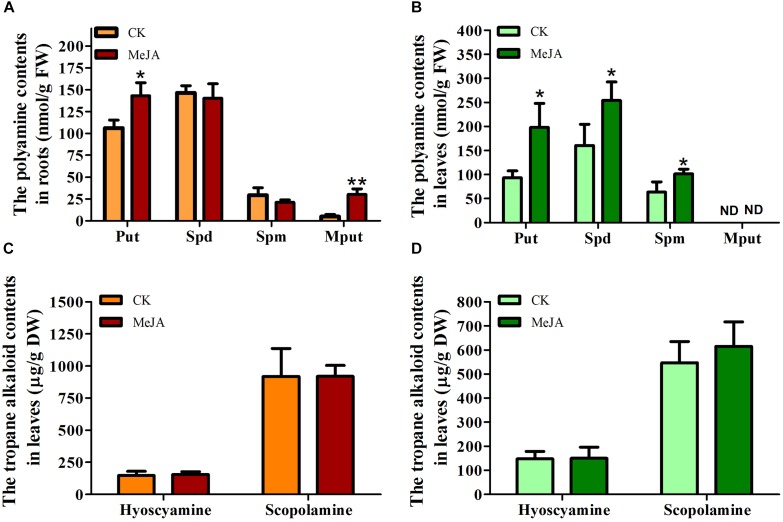
Concentrations of polyamines, *N*-methylputrescine, and tropane alkaloids in *Hyoscyamus niger* plants treated with methyl jasmonate (MeJA) and without (control, CK). The production of putrescine, spermidine, spermine, and *N*-methylputrescine in **(A)** roots and **(B)** leaves. The production of hyoscyamine and scopolamine in **(C)** roots and **(D)** leaves. ^∗^ and ^∗∗^indicate a significant difference at the levels of *P* < 0.05 and *P* < 0.01 (*t*-test), respectively. Vertical bars are means ± standard errors (*n* ≥ 3).

Since the above gene expression analysis revealed *HnODC* was upregulated by MeJA at the transcriptional level, it was reasonable to investigate ornithine-derived metabolite production in *H*. *niger* plants treated with MeJA (Figure 5). Specifically, we examined polyamines, *N*-methylputrescine, hyoscyamine, and scopolamine content in their roots and leaves. Putrescine production was significantly promoted in roots (Figure 5A) and leaves (Figure 5B), where its concentrations were respectively increased by 34.98 and 90.32% over the corresponding control plants. By contrast, the MeJA treatment did not affect spermidine and spermine production in the roots (Figure 5A), but it did elevate their concentrations significantly in leaves (Figure 5B). Roots of MeJA-treated plants had a 6.14-fold higher *N*-methylputrescine content (30.14 ± 6.52 nmol/g FW) relative to control roots (4.91 ± 2.54 nmol/g FW) (Figure 5A). In leaves of either MeJA-treated or control plants, *N*-methylputrescine was not detectable (Figure 5B). The production of hyoscyamine and scopolamine was also detected in *H*. *niger* roots and leaves, for which more scopolamine than hyoscyamine was found, consistent with former’s greater abundance than the latter in *H*. *niger* (Han Woo et al., 1995). However, hyoscyamine and scopolamine concentrations were not significantly changed in roots and leaves (Figure 5A,B) by MeJA, suggesting it did not significantly affect their production.

### Protein Purification and Enzymatic Assay

**FIGURE 6:**
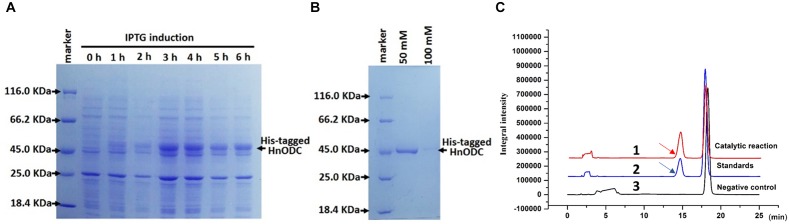
Expression, purification, and enzymatic assays of His-tagged HnODC. **(A)** Expression of His-tagged HnODC in engineered *E*. *coli*. **(B)** Purification of His-tagged HnODC using Ni^2+^-chelating resin and elution with 50 and 100 mM of imidazole. **(C)** HPLC detection of the product given by HnODC activity. The arrows indicate benzoylated putrescine. Line 1 in red represents the catalytic reaction of HnODC. Line 2 shows the reaction system with authentic benzoylated putrescine (as the positive control). Line 3 indicates the reaction system of boiled HnODC (as the negative control).

To determine its enzymatic activity, HnODC was expressed in *E*. *coli* to produce its recombinant proteins (Figure 6A). The HnODC enzymes could be readily obtained in the supernatants of lysed *E*. *coli*. Then, the His-tagged HnODC was purified using Ni^2+^-chelating resin column through elution with 50 mM of imidazole (Figure 6B). The molecular weight of recombinant HnODC was approximately 50 kDa (Figure 6A,B), consistent with its calculated molecular weight and similar to the molecular weight of other reported plant ODC enzymes. When the substrate, ornithine, was fed to HnODC, the products were successfully detected by HPLC with a retention time of 14.8 min (Figure 6C), which agreed with that of the standard (Figure 6C). In our negative controls, no product was detected when HnODC was boiled (Figure 6C). Together, these results show HnODC did catalyze ornithine to produce putrescine.

**FIGURE 7:**
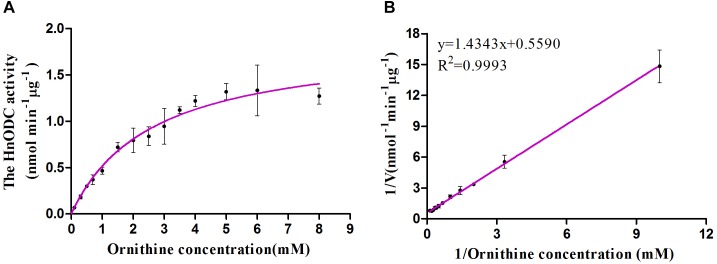
Michaelis–Menten curve of HnODC for ornithine **(A)** and Lineweaver-Burk plot for evaluation of Km and Vmax for HnODC **(B)**. Vertical bars are means ± SD (*n* = 3).

Enzymes catalyzing the same reaction in different organisms usually differ from each other in their enzymatic kinetics, such as affinity to substrate and catalytic efficiency. To obtain kinetics information on HnODC behavior, we derived its *K*_m_, *V*_max_, and *K*_cat_ values for ornithine: respectively, 2.62 ± 0.11 mM, 1.87 ± 0.023 nmol min^-1^ μg^-1^ and 1.57 ± 0.015 at pH 8.0 and at 30°C based on the Michaelis–Menten curve (Figure 7A). Notably, the *K*_m_ value of HnODC was higher than that of either EcODC (0.395 mM) or NgODC (0.56 mM) (Table 1), suggesting that HnODC had lower affinity to ornithine than EcODC and NgODC (Docimo et al., 2012). Since the *V*_max_ value of HnODC also exceeded that of EcODC and NgODC, its resulting *K*_cat_ value likewise greater. The *K*_cat_/*K*_m_ value, which expresses catalytic efficiency, was 599 M^-1^ s^-1^ for HnODC and thus higher corresponding values reported for EcODC (465 M^-1^ s^-1^) and NgODC (16.53 M^-1^ s^-1^) (Table 1). Lineweaver-Burk plot for evaluation of *K*_m_ and *V*_max_ for HnODC was shown in the Figure 7B. In sum, the kinetic analysis demonstrated HnODC had a lower affinity to ornithine but a much higher catalytic efficiency than displayed by EcODC and NgODC.

## Discussion

### HnODC Efficiently Converted Ornithine to Putrescine

Generally, all ODCs contain the conserved PLP-binding motifs composed of PFYAVKCN, and the GPTCD sequences, both of which are necessary for ODC activity (Coleman et al., 2003). Both of the motifs present in HnODC strongly suggest that it is a functional enzyme which catalyzes the decarboxylation of ornithine. Although the ODC sequences are similar to those of LDC, the phylogenetic analyses are able to distinguish between them. Since ODC and LDC each had its own clade in the phylogenetic tree, it has been suggested that they may have had common ancestors and evolved to different types of enzymes with slight modification (Bunsupa et al., 2012). Furthermore, the relatively high sequence similarity between the HnODC and other plant ODC proteins also suggest that HnODC should have similar functions to the other plant ODC enzymes.

Our biochemical assays confirmed that HnODC catalyzed the decarboxylation of ornithine to produce putrescine. HnODC had a lower affinity to ornithine than do the ODC enzymes of tobacco and coca tree, but its catalytic efficiency was found to be much greater that of ODCs of tobacco and coca tree. Particularly, HnODC showed about 36-fold increase in catalytic efficiency over NgODC. The low catalytic efficiency of tobacco ODC led it to be a limiting enzyme in nicotine biosynthesis, and consequently overexpression of yeast ODC enhanced the production of nicotine in transgenic tobacco (Hamill et al., 1990). In TA-producing plant species, the production of TAs was greatly reduced when ODC was inhibited by DFMO, suggesting that ODC might play a crucial role in TA biosynthesis (Nyman, 1994). High catalytic efficiency of HnODC facilitated the production of putrescine that entered the biosynthetic pathway of TAs. Due to higher catalytic efficiency of HnODC, it might be a better candidate for engineering the biosynthesis of putrescine-derived metabolites than the reported plant ODC enzymes.

### *HnODC* Was Highly Expressed in Roots and Up-Regulated by MeJA

Biosynthesis genes involved in the same pathway usually have similar tissue expression patterns. We found that in *Hyoscyamus niger* plants the TA biosynthesis genes of *HnPMT*, *HnTRI*, and *HnH6H* were expressed almost exclusively in secondary roots. Unlike them, *HnODC* was expressed in both roots and leaves yet its expression level was much higher in roots than in leaves. The different expression levels of *HnODC* likely reflect the changing metabolic demands for putrescine by root and leaf organs. Both polyamines and TAs were synthesized in roots and this required more production of its key precursor (putrescine); therefore, a high expression of *HnODC* in roots matched this requirement. The phytohormone, MeJA, could up-regulate the TA biosynthesis genes in a species-dependent way. *HnPMT* expression was dramatically elevated by MeJA in hairy root cultures of *H*. *niger* (Geng et al., 2018), while it was not affected by MeJA in *Atropa belladonna* (Li et al., 2014). *HnODC* was found strongly induced by MeJA in roots and leaves of the *H*. *niger* plants; however, neither *HnTRI* nor *HnH6H* was changed by MeJA at the transcriptional level. Hence, we conclude that MeJA positively regulated the TA-biosynthesis genes, including *HnODC* and *HnPMT*, though it did not regulate the TA-biosynthesis genes, such as *TRI* and *H6H*. Considering that ADC enzymes contribute to the putrescine biosynthesis, their expression was also detected. Unlike *HnODC* and TA biosynthesis genes with high or specific expression in roots, *HnADC1*/*HnADC2* was expressed in roots and leaves, with no significant difference. The two *ADC* genes were not responsive to MeJA treatment. Gene expression analysis suggested that the increased production of putrescine was mainly caused by the up-regulation of *HnODC*.

### MeJA-Induced Expression of *HnODC* Promoted the Production of Putrescine and *N*-Methylputrescine

Due to the MeJA-elevated expression of *HnODC*, putrescine—the product given by HnODC—production was significantly increased in roots and leaves, consistent with previously reported results that overexpression of ODC enhanced the production of putrescine in transgenic tobacco and rice (Lepri et al., 2001; Kumria and Rajam, 2002). In particular, we found *N*-methylputrescine production markedly promoted in roots when *H*. *niger* plants were treated with MeJA. This increased production could have been caused by both MeJA-induced expression of *HnPMT* and a greater supply of putrescine provided by MeJA-induced expression of *HnODC*. In the leaves with or without MeJA treatment, *N*-methylputrescine production was undetectable, obviously due to the lack of *HnPMT* expression in leaves; this results also indicates that *N*-methylputrescine synthesized in roots was hardly translocated to aboveground to leaf parts. Yet MeJA clearly promoted the production of spermidine and spermine in leaves, whereas their respective production in roots was not altered. Biosynthesis genes involved in TA pathway, such as *HnPMT*, *HnTRI*, and *HnH6H*, were not expressed in leaf, suggesting that putrescine was not metabolized into TA biosynthesis in leaf. However, putrescine was able to go into biosynthesis of spermidine and spermine in leaf. The increased putrescine production induced by MeJA resulted in more putrescine available for biosynthesis of spermidine and spermine in leaf. In roots, putrescine entered the biosynthesis of *N*-methylputrescine, spermidine, and spermine. MeJA-elevated *HnPMT* expression led to enhanced conversion of putrescine into *N*-methylputrescine in roots, and consequently the production of spermidine and spermine was at relatively stable levels. For hyoscyamine and scopolamine, the MeJA treatment did not affect their levels in roots and leaves, suggesting that the elevated expression of HnODC and HnPMT were not enough to promote the production of the two pharmaceutical TAs. Previously, overexpression tobacco *PMT* gene markedly promoted the *N*-methylputrescine production but did not enhanced the TA accumulation in root cultures of *H*. *niger* (Zhang et al., 2004). To conclude, the up-regulation of *HnODC* at transcriptional level was able to provide the precursors (putrescine and *N*-methylputrescine) at higher levels for TA biosynthesis and thereby promote polyamine production in *H*. *niger*.

## Author Contributions

TZ and ZL conceived and designed the study. TZ, CW, and FB performed gene cloning, expression analysis, and biochemical assays. FZ and GB performed bioinformatic analysis. CY and XL managed the plants. SL and MC detected metabolites. TZ and ZL prepared the manuscript. All the authors have read and approved the manuscript.

## Conflict of Interest Statement

The authors declare that the research was conducted in the absence of any commercial or financial relationships that could be construed as a potential conflict of interest.

## Footnotes

1 https://blast.ncbi.nlm.nih.gov/Blast.cgi
