# Supplementary material for: Metabolic Characterization of Hyoscyamus niger Ornithine Decarboxylase
Source: Front Plant Sci. 2019 Feb 27;10:229. doi: 10.3389/fpls.2019.00229 (PMC6400997; doi:10.3389/fpls.2019.00229)
Supplement: Supplementary file 1 [file Data_Sheet_1.docx]

**Supplementary Table S1** Primers used in this study.

| **Primers** | **Sequence 5'→3'** | | **efficiency (%)** | | **Tm (℃)** | | | **Purpose** |
| --- | --- | --- | --- | --- | --- | --- | --- | --- |
| qHnPGK-F | TCGCTCTTGGAGAAGGTTGAC | | | 97.8 | | 59.5 | internal reference gene in qRT-PCR analysis | |
| qHnPGK-R | CTTGTCGGCAATCACTACATCAG | | |  |  |  |  |  |
| qHnPMT-F | | CCTACTTACCCTACTGGTGTTATT | | 99.8 | | 58.4 | qRT-PCR analysis of *HnPMT* | |
| qHnPMT-R | | GCAAAAGATGGCAAAATAAAAGC | |  |  |  |  |  |
| qHnTRI-F | | TCACAAAGAAGATTACAAGA | | 99.5 | | 56.4 | qRT-PCR analysis of *HnTRI* | |
| qHnTRI-R | | GGAAGCAGAATAAAGAGAAACAG | |  |  |  |  |  |
| qHnH6H-F | | TTCCTCTTGAGCAGAAAGCAAAGC | | 98.4 | | 58.3 | qRT-PCR analysis of *HnH6H* | |
| qHnH6H-R | | CCTCATGGTCAACTTCCTCACTTCT | |  |  |  |  |  |
| qHnODC-F | | CGTCTTACTGTCTCTGGCGTCTC | | 99.1 | | 58.0 | qRT-PCR analysis of *HnODC* | |
| qHnODC-R | | GGTTGCGGCGGTTGTGAATTG | |  |  |  |  |  |
| qHnADC1-F  qHnADC1-R | | GCCACCGTCATCAAGTCAACTG  CTTCAACCCATCGCTCTTTACCC | | 99.5 | | 59.2 | qRT-PCR analysis of *HnADC1* | |
| qHnADC2-F  qHnADC2-R | | GGCAAGCAGCACCATCATC  GTACTACCACCAACAAGGAACC | | 99.1 | | 56.3 | qRT-PCR analysis of HnADC2 | |
| HnODC-F | | ATGTCCGGCCAAACAGTCATCG | |  | | 57.5 | Cloning of the coding  sequence of *HnODC* | |
| HnODC-R | | TCATCAGCTTGAATAAGCATAAGC | |  |  |  |  |  |
| pHnODC-F | | GC*GGATCC*ATGTCCGGCCAAACAGTCATCG | |  | | 58.0 | Constructing prokaryotic expression vector | |
| pHnODC-R | | GC*GAGCTC*TCATCAGCTTGAATAAGCATAAGC | |  |  |  |  |  |

The restriction sites were underlined.

**
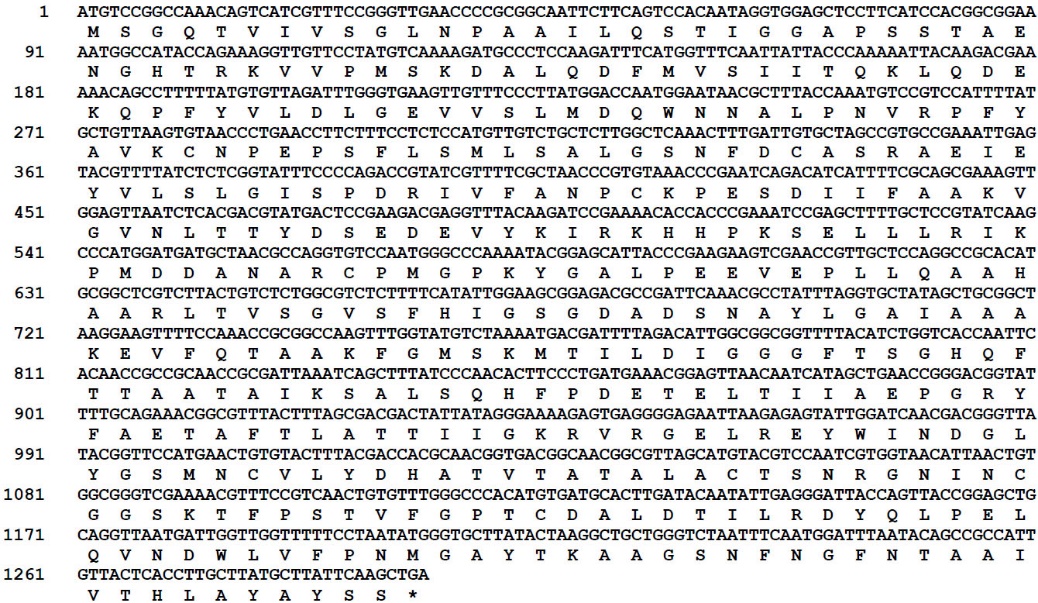
**

**Supplementary Figure S1** The coding sequence of *HnODC* and its deduced amino acid sequence. The nucleotide sequence and its corresponding amino acid sequence are shown in capital letters. ∗ represents the stop codon.


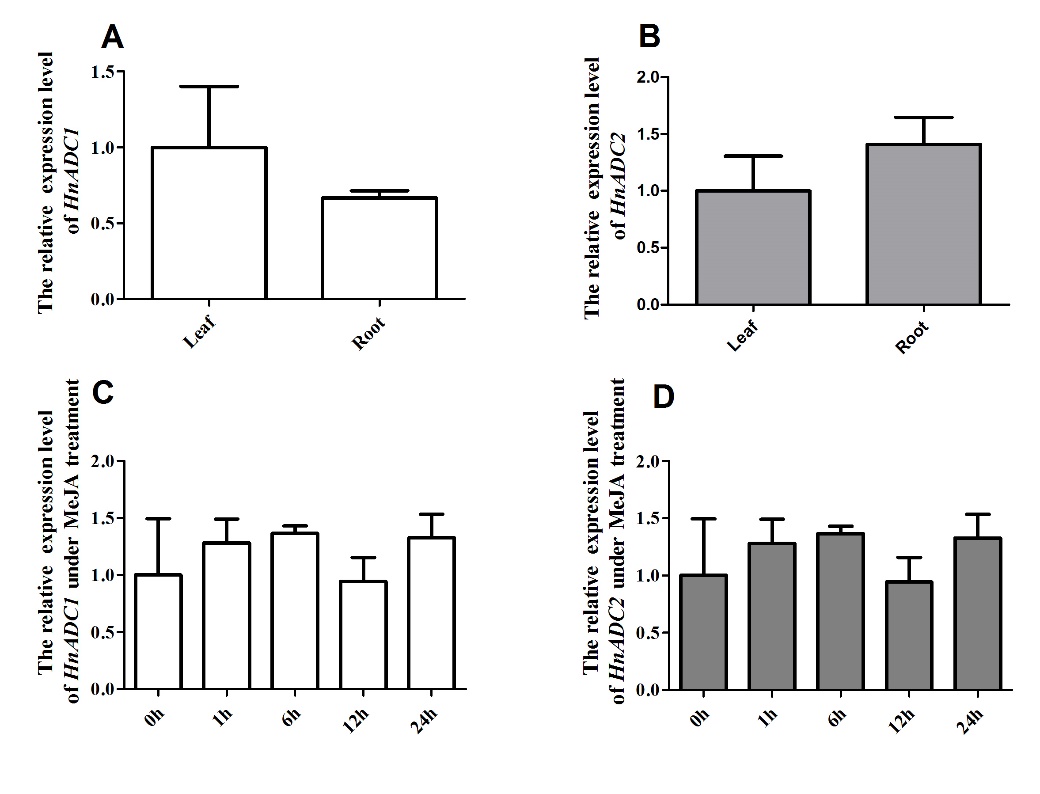


**Supplementary Figure S2** Tissue profiles of *HnADC1/HnADC2* genes and their expression patterns in *Hyoscyamus niger* plants treated with MeJA for 0 to 24 h. Overall expression levels in roots and leaves of **(A)** *HnADC1*, **(B)** *HnADC2*. Expression of **(C)** *HnADC1*, **(D)** *HnADC2*, according the duration of treatment with methyl jasmonate. Vertical bars are means ± standard errors (n ≥ 3). ** indicates a significant difference at the level of P < 0.01 (*t* test).
